# Reducing Negative Outcomes of Online Consumer Health Information: Qualitative Interpretive Study with Clinicians, Librarians, and Consumers

**DOI:** 10.2196/jmir.9326

**Published:** 2018-05-04

**Authors:** Reem El Sherif, Pierre Pluye, Christine Thoër, Charo Rodriguez

**Affiliations:** ^1^ Department of Family Medicine McGill University Montreal, QC Canada; ^2^ Department of Social and Public Communication University of Quebec in Montreal Montreal, QC Canada

**Keywords:** consumer health information, internet, professional-patient relations, qualitative research

## Abstract

**Background:**

There has been an exponential increase in the general population’s usage of the internet and of information accessibility; the current demand for online consumer health information (OCHI) is unprecedented. There are multiple studies on internet access and usage, quality of information, and information needs. However, few studies explored negative outcomes of OCHI in detail or from different perspectives, and none examined how these negative outcomes could be reduced.

**Objective:**

The aim of this study was to describe negative outcomes associated with OCHI use in primary care and identify potential preventive strategies from consumers’, health practitioners’, and health librarians’ perspectives.

**Methods:**

This included a two-stage interpretive qualitative study. In the first stage, we recruited through a social media survey, a purposeful sample of 19 OCHI users who had experienced negative outcomes associated with OCHI. We conducted semistructured interviews and performed a deductive-inductive thematic analysis. The results also informed the creation of vignettes that were used in the next stage. In the second stage, we interviewed a convenient sample of 10 key informants: 7 health practitioners (3 family physicians, 2 nurses, and 2 pharmacists) and 3 health librarians. With the support of the vignettes, we asked participants to elaborate on (1) their experience with patients who have used OCHI and experienced negative outcomes and (2) what strategies they suggest to reduce these outcomes. We performed a deductive-inductive thematic analysis.

**Results:**

We found that negative outcomes of OCHI may occur at three levels: internal (such as increased worrying), interpersonal (such as a tension in the patient-clinician relationship), and service-related (such as postponing a clinical encounter). Participants also proposed three types of strategies to reduce the occurrence of these negative outcomes, namely, providing consumers with reliable OCHI, educating consumers on how to assess OCHI websites, and helping consumers present and discuss the OCHI they find with a health professional in their social network or a librarian for instance.

**Conclusions:**

We examined negative outcomes associated with using OCHI from five complementary perspectives (consumers, family physicians, pharmacists, nurses, and health librarians). We identified a construct of OCHI use–related tension that included and framed all negative outcomes. This construct has three dimensions (three interdependent levels): internal, interpersonal, and service-related tensions. Future research can focus on the implementation and effectiveness of the proposed strategies, which might contribute to reducing these tensions.

## Introduction

### Online Consumer Health Information

Online consumer health information (OCHI) is defined as information on health and diseases that is created for and directed to the general public [1]. General health information is available in written, audio, and video formats and freely accessible on government sites, professional organizations’ websites, health journals, online forums, and blogs, among other sources. Moreover, consumers actively use social media to facilitate self-care, as well as being passively exposed to OCHI posts being shared by their social network through social media platforms such as Facebook [2,3].

American surveys of representative samples of the population have shown that the use of OCHI has increased dramatically over the last decade, and the internet is now the most popular source of consumer health information, whereas the use of other sources has decreased [4,5]. Reasons for this are the sheer volume of readily accessible health information available online, the increased engagement of people in their own health and self-care, and decreased access to health care services [6-8].

Increased access to OCHI is generally associated with increased consumer engagement in their own health care, increased empowerment of themselves and their families, and improved health outcomes [9-11]. Indeed, one of the most common ways consumers use OCHI is for consultation with health practitioners, for engagement in their own health care, compliance with or modification of management plan, or support of relatives or friends with health conditions [12]. There may, however, be some unintended negative consequences that are poorly understood and perhaps under reported [13,14]. With the increasing amount of OCHI available and the exponential increase in OCHI use, these negative consequences may also increase. The aim of this study was to identify and understand these negative outcomes from the viewpoint of primary care consumers and practitioners and try to find ways to reduce them.

### Literature Review

Although there is evidence available on OCHI use and its outcomes in primary care, few studies have focused on the possible negative outcomes. In 2015, we conducted a systematic mixed studies review that examined outcomes associated with OCHI in primary care [15]. This review included 65 studies [15]. Although most of these studies reported positive outcomes (eg, reduced worries, increased satisfaction with health care services, increased involvement in decision making, and improvement of health), 23 studies described negative outcomes associated with using OCHI from either a physician or a patient perspective. One of the most well-known negative outcomes mentioned by both parties is increased anxiety, sometimes referred to as *cyberchondria* by researchers. Although physicians perceive this anxiety as *excessive or overestimated*, it is a reported consequence of looking for and using OCHI [16,17]. Another commonly reported outcome found in our review is deterioration in the patient-clinician relationship, especially after the patient shares the retrieved OCHI with a clinician [14]. A third outcome is the effect of using OCHI on the health care system resources, for example, leading to longer, unnecessary encounters with the family physician [18].

Most of the studies in our review were set in an oncology or public health setting and focused on specialized information (as opposed to general) or specific patient populations (eg, pregnant women) [19]. This is problematic as not all results from a specialist setting or tertiary health care population are transferrable to a primary care setting.

**Figure 1:**
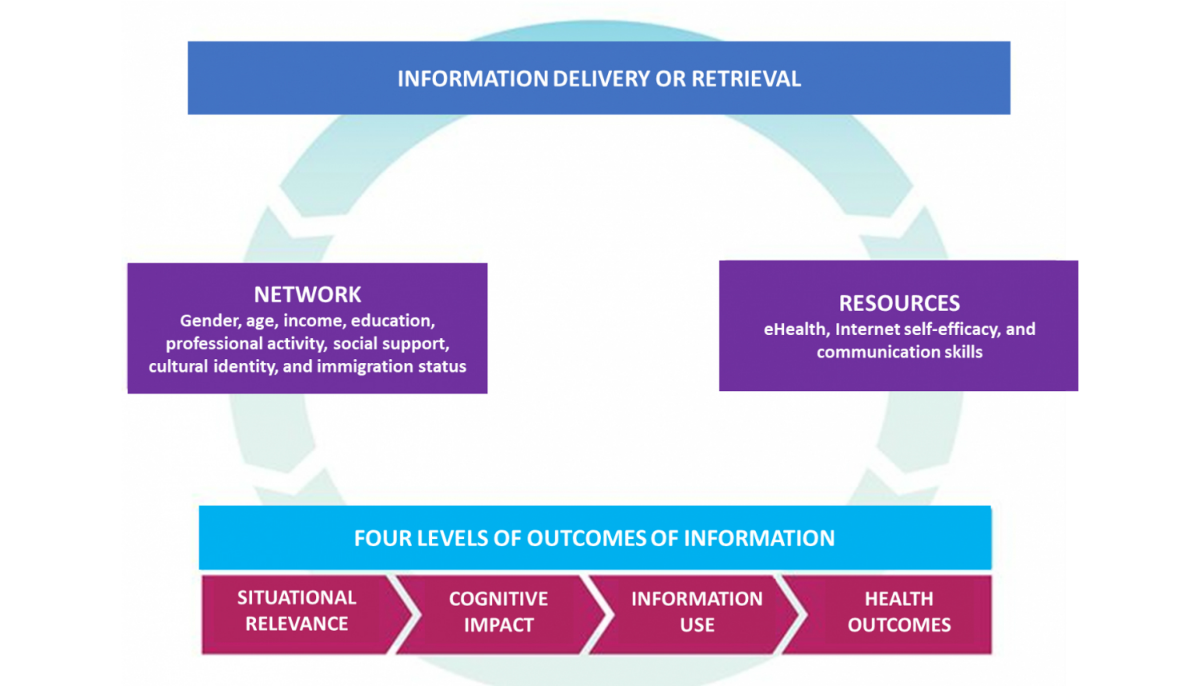
Conceptual framework.

### Objectives

The research question that guided this study was as follows: what are the negative outcomes of OCHI, and how can they be reduced? More specifically, we aimed to reach the following two objectives: (1) to identify and understand the meaning of the negative outcomes associated with OCHI use from consumers’, health practitioners’, and librarians’ viewpoints in primary care and (2) to report means to reduce these negative outcomes proposed by consumers, health practitioners, and librarians.

### Conceptual Framework

Our conceptual framework (Figure 1) [20] includes four levels of outcomes of information delivery and retrieval [12,21]: situational relevance, cognitive impact and use, and health or well-being outcomes of information. These levels are defined in relation to a specific information-seeking context: eg, a Web page used in a patient-clinician encounter. This framework was used to develop the interview guides and guided the deductive data analysis.

## Methods

### Study Design

We conducted a two-stage exploratory interpretive qualitative study that allows researchers “to obtain straight and largely unadorned answers to questions of special relevance to health care providers (HCPs) or policy makers” [22]. Methods and results are reported using the consolidated criteria for reporting qualitative studies [23]. We received ethical approval from the McGill University Faculty of Medicine’s Research Ethics Office (institutional review board) before we started recruitment

### Stage 1—Consumers

#### Sampling

We used a purposive sampling strategy to find participants who had experienced negative outcomes after using OCHI for themselves. Using a short online survey on SurveyMonkey (Multimedia Appendix 1) as a recruitment tool on social media platforms (Facebook and LinkedIn), we found a broad range of potential cases [24]. Some of the reported benefits of using Facebook to recruit participants include “reduced costs, shorter recruitment periods, better representation, and improved participant selection in young and hard to reach demographics” [25].

We received 148 completed surveys; 75 respondents indicated that they had experienced both positive and negative consequences of using OCHI and agreed to be contacted. These respondents were emailed a consent form by order of response (five per day) until data saturation was reached.

#### Data Collection

To accommodate the participants’ time constraints and geographic dispersion, we used semistructured telephone interviews. Telephone interviews encourage a more explicit description of the participant’s emotional experiences because of the absence of visual cues [26]. We developed an interview guide based on our conceptual framework, pilot-tested it for clarity, and modified it accordingly (Multimedia Appendix 2). Participants were asked to elaborate on their negative experience with OCHI use by telling their *story* and what factors they believe may have led to these outcomes. They were also asked what they think could have been done to prevent these outcomes. The 10-20 min interviews were recorded and conducted from November 2015 to February 2016. Interviews were transcribed verbatim (using pseudonyms to protect identities), and the transcripts were imported into the NVivo 11 software package (QSR International Pty Ltd, Victoria, Australia) for qualitative data analysis. Interviewing continued until data saturation was reached after 19 participants [27].

#### Data Analysis

A deductive-inductive analytical approach was adopted for coding [28]. We created a coding manual of the types of use and types of outcomes, both positive and negative, and the codes were discussed with the team until a consensus was reached. The codes were then progressively clustered into major themes. We also performed a secondary analysis of the interview transcripts using a story telling technique [29]. This involved viewing the interview transcript through *multiple lenses* and developing interpretive stories based on those lenses [30]. These stories, or vignettes, were created to represent each of the different types of negative outcomes identified in the literature review and the interviews. In stage 2 of this study, these stories were used to introduce the topic to the health practitioners and health librarians.

### Stage 2—Health Professionals

#### Sampling

In stage 2, we selected seven health practitioners (three family physicians, two registered nurses, and two community pharmacists) and three health librarians because they are considered a primary source of health information for their patients [31]. These participants are considered key informants on the measures that could be taken to prevent negative consumer outcomes [27].

We used a purposeful sample of these key informants in Montreal and Ottawa. Using personal contacts in the Department of Family Medicine and the School of Information Studies at McGill, the Herzl Family Medicine Clinic, the Jewish General Hospital, and the Canadian Pharmacists Association of Canada, we invited primary care practitioners and health librarians to participate by email.

#### Data Collection

We conducted in-person semistructured interviews, 45 and 60 min in length, from March 2016 to April 2016. All the interviews were conducted at the participant’s office (either in a clinic or hospital). We developed the interview guide based on our conceptual framework, pilot-tested it for clarity, and modified it accordingly (Multimedia Appendix 2). At the beginning of the interview, three vignettes created from the stage 1 interviews were presented to the stage 2 participants (Textbox 1).

**Vignettes presented at the start of the interview with health care practitioners and health librarians.**

**Vignette 1**

A young 22-year-old woman who identifies herself as a “bit of a hypochondriac” usually goes online to look for health information when she has multiple symptoms and she is unsure if they are related or not. She uses information to decide if she needs to see a doctor or not.

On one occasion, she had pain near her ribs and pain with “breathing.” After checking online, she found “scary” diagnoses of similar symptoms and decided to go to the emergency room. After waiting there for a few hours, she was told it was nothing and went home. This happened a few times.

She feels that if there was more specific information online, or lists of the “most common diagnoses” for each symptom, she would worry less about her online findings.

**Vignette 2**

Mark is a 32-year-old man who usually looks for health information online when he has a new unfamiliar symptom. He uses information to decide whether (or not) he needs to see a doctor and to find possible explanations for his symptoms. The information helps him reflect on his lifestyle and determine if there are any changes he needs to make.

On one occasion, after suffering from abdominal pain for months, his family physician requested an ultrasound. During the ultrasound, and after some probing from Mark, the ultrasound technician suggested it may be a polycystic kidney causing his pain. As his follow-up appointment with the specialist was weeks away, Mark decided to do as much research on the topic as possible, which led to increasing anxiety over this diagnosis. Eventually at his appointment, the specialist diagnosed him with a failed kidney and not polycystic kidneys, which while severe, was still a relief for Mark.

Although in this case Mark blames the technician for making an unfounded diagnosis, he feels doing so much research on the topic allowed him to have an educated discussion with the specialist during his appointment.

**Vignette 3**

Sarah is a 26-year-old woman who was diagnosed with epilepsy. Her doctor prescribed Depakene as the best treatment, and she started using it. Then she started getting side effects from this medication.

After looking online for health information on this issue, she found that there were complementary and alternative treatments for epilepsy such as reiki and yoga, as well as herbal remedies, dietary supplements, and homeopathic treatments. She read testimonies by other people who had used these alternatives other than the Depakene and decided to perhaps try following them instead.

She feels that the information she found was biased, “you find what you look for.” She admits that while looking for information, she might have valued a lot less information that said she needed to take the Depakene and valued more heavily information that said epilepsy was potentially manageable alternative and homeopathic remedies.

The vignettes offered the practitioners and librarians with an opportunity to reflect on the negative outcomes resulting from OCHI use and refreshed their memories of their own experiences. They were then asked to give their opinion on the situations described in the vignettes, as well as elaborate on their own experience with consumers or patients using OCHI. Finally, practitioners were asked what strategies they used to prevent or alleviate negative outcomes in their practice, whereas health librarians were asked what strategies they believe should be used to prevent negative outcomes. The interviews were conducted, transcribed, and analyzed two at a time [27]. No new codes emerged during the analysis of the 9th and 10th interviews, so data collection stopped.

#### Data Analysis

Using NVivo 11, we coded the transcripts for types of negative outcomes and preventive measures. Similar to the first stage, a deductive-inductive analytical approach was adopted [27]. We created a coding manual of the types negative outcomes and preventive measures, and the codes were discussed with the team until a consensus was reached. The codes were then progressively clustered into major themes.

## Results

### Overview of the Results

A description of the participants of both stages is presented in Tables 1 and 2. In both stages, many positive outcomes of OCHI were found: OCHI allows consumers to be informed and involved in their health care. According to participants, consumers have different strategies for finding and assessing OCHI and that factors such as health literacy and access to an HCP influence the type of outcome that occurs. Participants of both stages described similar negative outcomes such as increased worrying and postponing a health care visit. Preventive strategies mentioned in both stages included providing reliable sources of OCHI, teaching consumers to properly assess OCHI found, and discussing OCHI with someone else.

### How Consumers Find, Understand, and Use Online Consumer Health Information

Consumers described their motivations and strategies for searching for OCHI, their understanding of the information they found, and how they used it. The identified themes and subthemes are described in Table 3. In summary, participants would most commonly search for information for themselves or for someone else by entering their symptoms into a search engine (“Googling their symptoms”). They had different ways of deciding the credibility of a website, and for the most part, just wanted more information on an issue, though some participants used OCHI to decide whether or not to book an appointment with a HCP or stop a medication, as illustrated in the following quote:

I've looked up stuff like side effects of birth control pills if I'm worried or more emotional, I'll see if that is one. I've actually gone off [pills] because of that.

Jenny

**Table 1:** Participants in stage 1.

Pseudonym	Gender	Age group (years)
Alan	Male	18-24
Betty	Female	18-24
Cara	Female	18-24
Dina	Female	18-24
Ella	Female	18-24
Fred	Male	18-24
Gina	Female	18-24
Harry	Male	25-34
Isabel	Female	45-54
Jenny	Female	18-24
Karen	Female	18-24
Lara	Female	25-34
Mariah	Female	18-24
Nathan	Male	18-24
Pamela	Female	18-24
Rita	Female	18-24
Sarah	Female	18-24
Tamara	Female	18-24
Vanessa	Female	25-34

**Table 2:** Participants in stage 2.

Alias	Profession	Work environment
Pharmacist #1	Clinical pharmacist	A family medicine clinic attached to a teaching hospital in Montreal.
Pharmacist #2	Community pharmacist	A community pharmacy and a family medicine clinic in Ottawa.
Doctor #1	Family physician	An academic hospital and a walk-in clinic in Montreal.
Doctor #2	Family physician	An academic hospital and a walk-in clinic in Montreal.
Doctor #3	Family physician	A family medicine clinic in Ottawa.
Librarian #1	Health librarian	A hospital health sciences library in Montreal.
Librarian #2	Health librarian	A hospital health sciences library in Montreal.
Librarian #3	Health librarian	A children’s hospital health sciences library in Montreal.
Nurse #1	Nurse	An academic hospital in Montreal.
Nurse #2	Nurse practitioner	A family medicine clinic affiliated with an academic hospital in Montreal.

**Table 3:** Finding, understanding, and using online consumer health information (OCHI).

Theme and subtheme		Example quote
**1. Motivation for searching for health information online**		
	1.1 Searching for information for themselves	“When it is something that I cannot explain, like I have multiple symptoms and I don’t know if all these symptoms are related, unrelated...” [Alan]
	1.2 Hypochondria	“I’m a little hypochondriac, I mean literally last night I was feeling nauseous, so I started to Google, so anytime I’m feeling an odd symptom...anytime I feel something is abnormal and I’ll look up those symptoms to see if I have anything, from nausea and headaches to weird circulatory feelings.” [Rita]
	1.3 Searching for information for someone else	“Last time I looked up stuff online was for my grandfather. He suffered from Parkinson’s...and we were looking for alternatives.” [Nathan]
**2. Strategies for searching for information online**		
	2.1 Using a search engine (Google)	“I usually Google either my symptoms if I don’t know what it is, or if I have an idea of what it might be then I'll Google that.” [Betty]
	2.2 Using a renowned medical website	“I just Google but the ones I usually end up in are WebMD or mayo clinic, I think if you Google something those are the first ones that show up anyway.” [Rita]
	2.3 Using websites or forums with patient experiences	“...there are a lot of useful forums where experienced marathoners have training advice, stuff like that. When to do icing or heat, which one is better than the other.” [Ella]
	2.4 Strategies for evaluating OCHI websites	“I usually avoid sites that are trying to sell you stuff or that anyone can edit.” [Tamara]
**3. Making sense of the information**		
	3.1 Understanding the information found	“I understand it, I might have to do further research for specific terms, but overall I understand what they’re saying.” [Alan]
	3.2 Gaining general knowledge without answering a specific question	“Sometimes you don’t know what is wrong or right and each case is different as well, so you have an idea globally, but you don’t really have the answer I guess.” [Mariah]
	3.3 Not finding the answer to a specific health question	“No. I would have a symptom and it would usually end with me convincing myself that I had some sort of terminal illness.” [Cara]
	3.4 How health literacy influences understanding	“No, I can usually understand it. I feel like I may be more science and health literate than a lot of people since I have a Bachelor’s degree in Science.” [Betty]
**4. Decision making after finding relevant OCHI**		
	4.1. Deciding whether or not to book a medical appointment	“I wouldn’t say immediately but when I have a recurring kind of problem, so I'll look at it probably before calling the doctor and making an appointment.” [Isabel]
	4.2 Postponing a medical appointment because of limited access	“It’s not even that, it’s that you have to wait so long now to get an appointment that if I can home remedy it that’s how I sort of look at it.” [Isabel]
	4.3 Making a health care decision	“Usually if it’s something like I can change what I’m eating, I follow if it doesn’t seem to extreme or too hard to do. If it’s something that seems a bit ridiculous then...” [Tamara]
	4.4 Stopping a medication	“I’ve looked up stuff like side effects of birth control pills if I’m worried or more emotional, I’ll see if that is one. I’ve actually gone off [pills] because of that.” [Jenny]
	4.5 Discussion in a physician encounter	“Some things I’ll bring up when seeing my physician and get their advice on it.” [Dina]
	4.6 To confirm a physician’s diagnosis	“Yes, I have symptoms and look them up and if I find what I think it is I go to the doctor and I’ll let the doctor suggest on their own but I’ll kind of suggest that this what I think it could be, could you confirm that for me or not?” [Sarah]

### Health Care Practitioners’ and Health Librarians’ Experience With Online Consumer Health Information

Stage 2 participants described their general opinion of OCHI, the types of OCHI they had encountered, and factors they believed influenced the outcomes of OCHI use. The identified themes and subthemes are described in Table 4.

All the participants had seen their patients, their clients, and their friends and family use OCHI and had used OCHI themselves in their own health care. They believed it was a permanent fixture and an inevitable presence in the health care system, as illustrated in the following quote:

For patients, “I saw this online” is the new “my friend told me,” which I still see a lot with the elderly, although even the elderly are going online.

Pharmacist #2

Participants had experiences with different formats and topics of OCHI depending on their profession, the location of their practice, and the types of patients or consumers they saw. Physicians reported dealing with diverse topics (eg, medications and their side effects, medical conditions, and diagnostic tests) from various sources (reviewed online medical resources, patient forums, blogs, etc). Nurses, on the other hand, are traditionally more involved in patient education, and therefore tend to be more exposed to OCHI on general health information rather than specific health conditions. Pharmacists were more exposed to OCHI covering medications and their side effects and herbal products or supplements. Health librarians are traditionally health information providers, so are not necessarily exposed to patients presenting them with OCHI. They were, however, very familiar with the different sources of OCHI available, specifically patient forums, and were aware of its pros and cons.

A recurrent theme during the interviews was the OCHI related to alternative and complementary medical treatments and therapies. Participants mentioned some specific examples where patients had read of an unconventional treatment for their condition online and wanted to find out if it was a viable alternative. Another theme that was brought up during the interviews was the antivaccine movement, and participants mentioned interactions with people about vaccinations for which there is conflicting OCHI.

### Negative Outcomes of Online Consumer Health Information

We identified three negative outcomes of OCHI based on participants’ use of it (Table 5). First, increased worrying as a result of finding “scary” or worse-case-scenario information that might or might not be relevant to their symptoms. Second, tension in the relationship with a family member because the latter’s use of potentially harmful OCHI. Finally, postponing seeking medical help for a health problem, or to ignore their health problem altogether.

**Table 4:** Health care practitioners’ and health librarians’ experience with online consumer health information (OCHI).

Theme and subtheme			Example quote
1. General opinion of participants on OCHI			“And for patients, ‘I saw this online’ is the new ‘my friend told me,’ which I still see a lot with the elderly, although even the elderly are going online.” [Pharmacist #2]
**2. Types of OCHI**			
	2.1 General health information	“I once saw a patient who had a dry cough and nothing else and came into an appointment because her friend had posted on Facebook that she had pneumonia.” [Doctor #3]	
	2.2 Forums and patient-sourced information	“There are a ton of forums online people talking about their personal experiences...you don’t get that from your health professional, they don’t know what it’s like to live with a condition. So, it can be very helpful to see other people’s experiences and it may give your ideas for alternative treatments.” [Librarian #1]	
	2.3 Alternative medicine information	“I had a patient who was relatively healthy but had high blood pressure that he treated with valerian root he had read about online...and so I looked it up and there was no real evidence for its effect on blood pressure.” [Nurse #1]	
	2.4 Antivaccination information	“It’s extremely frustrating because a lot of this antivaccine stuff is focused on really small risks and you have to acknowledge there might be risks and people tend to fixate on them, like there is mercury in vaccines, yes but there’s mercury in food. So, it can be extremely time consuming to combat that. I think that topic is the biggest and most harmful.” [Pharmacist #2]	
**3. Factors influencing outcomes**			
	3.1 Individual characteristics	“Definitely low health literacy but there also really well-educated people who don’t have a health background and can be quite susceptible to the alternative medicine stuff. In another clinic where I work we see a lot of new immigrants, a lot of them Arabic speaking, I can’t work with them as much.” [Pharmacist #2]	
	3.2 Information avoidant personalities	“I think if you know your patient and kind of know they’re the type who would basically somaticize every side effect you’re not going to go over them in as much detail, you will sort of down play them.” [Nurse #1]	
	3.3 Access to health care services	“Just that in Montreal, I don’t know exact numbers, but around 30-40% of people don’t have a family doctor, and the more vulnerable you are the more your access to good medical care decreases so I think that yes there are flaws to internet usage to access health care, but in a system where person-to-person health care is not good or easy to access, it may be the only resource that many people have available to them.” [Doctor #2]	

**Table 5:** Negative outcomes of online consumer health information (OCHI).

Stage and subtheme		Example quote
**Stage 1: consumers**		
	Increased worrying	“Sometimes it is anxiety inducing. If you can’t find something that’s a good match for what symptoms you’re having or if you find something that is a good match that isn’t so pleasant.” [Betty]
	Tension with family members	“One of my aunts takes online health info way too far, and everything online, she follows, it doesn’t matter where it’s from which is horrible because the internet has all sort of things...for 5 years my cousin’s life had all the random health natural remedies online, never doctors, it was so bad. It was disturbing when we found that when he would have an infection she wouldn’t take him to a doctor but make him drink honey...This situation caused stress between family members worried about the information she used.” [Vanessa]
	Postponing (not seeking help for) a health problem	“All my symptoms match a virus going around I read about it and I thought it will, blow over in a week, I don’t need to miss class to go to a doctor’s appointment and then I ended up going to the doctor and it ended up being an ear infection and a sinus infection and it turned into 2 months of being miserable...” [Ella]
**Stage 2: practitioners and librarians**		
	Increased worrying	“Yeah, I think so, she was worried, she took time off work to come in to see me, and she waited in the waiting room for a while. So, I have to take her worry seriously. This applies to many patients I see, where there are no actually worrisome symptoms, if they had waited a few days whatever they had would have gone away on its own. But they had read something online either after Googling their symptoms or after accidentally stumbling on a piece of online information through social media for example, and they worry they might have that.” [Doctor #3]
	Spending money on nonbeneficial products	“A lot of the herbal and complementary and alternative therapy stuff, the biggest harm to a lot of people is that it costs money and might not work... BP: I think the main consequence is that they can’t afford, it’s common for people who are poor to have poor literacy so will believe all this stuff they read online or Dr. Oz, so they end up spending money that they shouldn’t be spending.” [Pharmacist #2]
	Tension in the provider-patient relationship	“I want them to know that I’m aware of it, that I’m not ignorant, because a lot of time this OCHI can undermine their trust in your ability and your competence and they will say why didn’t you tell me about this? And sometimes the reason we haven’t told is because we think that it will just scare them which is true, and we do.” [Nurse #1]
	Nonadherence to management plan	“I think one of the biggest ones, the area I’ve had most problems with is mental health, it’s a huge issue and affects a particular anxiety, a patient who is going through a lot of problems unfortunately the internet and their ability to get information is a major block to being treated. They would look up the side effects of the medications because they are more suggestible, experience every side effect of the medication and eventually stop it.” [Nurse #1]
	Postponing seeking medical help	“We had a gentleman come in here [health library] and he was looking for information, and he started discussing what was wrong with him and saying he felt numbness in his leg and I said immediately let me get you a wheelchair and transport you to the emergency room. He was asking me for info about something that I clearly couldn’t solve, and part of my job is identifying when someone comes to me and saying you should go see a doctor or go to the emergency room.” [Librarian #2]

Similar to the first stage, increased worrying was a negative outcome found in stage 2 (Table 5). This could stem from reading reliable but nonrelevant information, from reading too much information, or from finding incorrect information from unreliable websites. It was advice on these latter websites that led to the second negative outcome: the purchase of useless or potentially harmful medications online. A third negative outcome is the breakdown of trust in the patient-clinician relationship from, for example, clinicians not validating patients’ information-seeking efforts. Moreover, finding information that contradicted that provided by the health practitioner also led to a breakdown in trust and lowered adherence to the management plan.

### Tension: A Comprehensive and Meaningful Construct

On further examination of these outcomes, they appeared to fall under one main theme of *tensions*, with three dimensions, depending on who or what was being affected by OCHI use: internal, interpersonal, and service-related (summarized in Table 6).

### Strategies for Reducing Online Consumer Health Information Negative Outcomes

Stage1 participants identified strategies that they used or believed would be helpful, such as managing expectations when searching for health information online, using reliable OCHI sources provided or reviewed by health practitioners, and discussing the information found with a health practitioner to validate its reliability and relevance to their health question. These strategies are presented in Table 7.

**Table 6:** Online consumer health information (OCHI) tensions.

Levels	Stage 1: consumers	Stage 2: practitioners and librarians
Internal tensions	Increased worrying	Increased worrying; Spending money on nonbeneficial products
Interpersonal tensions	Tension with family members	Tension in the provider-patient relationship
Service-related tensions	Ignoring (not seeking help) for a health problem	Nonadherence to management plan; Postponing seeking medical help

**Table 7:** Strategies for reducing negative outcomes of online consumer health information (OCHI).

Stage and subthemes		Example quote
**Stage 1: consumers**		
	Be aware of limitations of OCHI	“You have to be careful, when you do a Google search you get a ton of stuff there and sometimes rewording your search you get different things, so you want to be reading the same thing and not doing something that could do more damage than good.” [Isabel]
	Reliable and relevant sources of OCHI	“I think there are already doctors online, but I don’t know maybe something more precise because Web MD can be precise but it’s not that precise, like you can have just normal back pain and it will direct you to kidney failure.” [Lara]
	Follow physician-provided OCHI or search parameters	“...but also, if there is a 2-week delay between getting a scan and seeing the professional about something that is serious, you should be provided with, you know, here are search parameters that you should look up that are neutral and that might give you content to reflect on so that you have an informed discussion with your doctor…” [Harry]
	Discuss OCHI with physician, telehealth, or members of social network	“I keep in mind that it’s on the internet, so if I was really stressed I would go talk to a real person. I am skeptical of the information so if I was worried I would go talk to a doctor.” [Jenny]
**Stage 2: practitioners and librarians**		
	Provide reliable sources of OCHI	“Look it’s there, so instead of resisting it, let’s provide high quality alternatives so we have a little more control.” [Librarian #1]
	Teach people how to evaluate OCHI	“I think that if more health care providers used the approach of showing people where they look for info and pointing out potential issues with their sources and that is very effective, but it is time consuming.” [Pharmacist #2]
	Discuss OCHI during a clinical encounter	“I’ve mostly had a more positive experience just by being open and discussing it.” [Nurse #1]
	Nonadherence to management plan	“I think one of the biggest ones, the area I’ve had most problems with is mental health, it’s a huge issue and affects a particular anxiety, a patient who is going through a lot of problems unfortunately the internet and their ability to get information is a major block to being treated. They would look up the side effects of the medications because they are more suggestible, experience every side effect of the medication and eventually stop it.” [Nurse #1]
	Handling OCHI on alternative treatments or nonconventional therapies	“For the third vignette, we certainly don’t discourage exploration into complementary and alternative treatments, we have an excellent evidence based database we can search that have knowledge synthesis of the research that shows whether a given alternative treatment is actually effective, so we could have looked at maybe different remedies to show if there is any solid evidence and if it actually works and maybe there’s definitely bias.” [Librarian #1]

**Table 8:** Preventive strategies.

Strategies	Stage 1: consumers	Stage 2: practitioners and librarians
Before OCHI^a^ search: providing reliable sources	Be aware of limitations of OCHI; Reliable and relevant sources of OCHI; Follow physician-provided OCHI or search parameters	Provide reliable sources of OCHI
During the search: teaching consumers how to evaluate OCHI sources	Using OCHI sources that are of good quality	Teach people how to evaluate OCHI sources
After finding relevant OCHI: discussing the information found	Discuss OCHI with physician, telehealth, or members of social network	Discuss OCHI during a clinical encounter; Handling OCHI on alternative treatments or nonconventional therapies

^a^OCHI: online consumer health information.

Similar strategies were also proposed by stage 2 participants to reduce the occurrence of negative outcomes. First, they recommended providing reliable OCHI sources. Second, teach consumers how to assess websites, either during an appointment or by referring them to online resources. Third, encourage consumers to discuss the OCHI with an HCP during a clinical encounter to validate its reliability and relevance. Our findings suggest this may be important for OCHI about alternative and complementary therapies, where it is important to explain to consumers the difference between regulated and unregulated therapies, as well as how to assess the reliability of the information found. Finally, our results suggest that because of the frequency of OCHI use, HCPs should be trained to deal with patients who bring OCHI to a clinical visit. These strategies are presented in Table 7.

Participants also suggested discussing OCHI with health librarians as they are well situated to provide reliable OCHI sources, teach consumers how to evaluate websites, help consumers prepare the information to discuss during a clinical encounter, and find reviewed evidence on complementary and alternative therapies.

In summary, many preventive strategies were proposed by participants to reduce the occurrence of negative outcomes, as shown in Table 8. These include providing reliable OCHI sources before consumers start the search, teaching consumers to evaluate websites, and encouraging consumers to validate the information’s reliability and relevance with an HCP.

## Discussion

### Principal Findings

Our findings confirm that OCHI is a part of daily routines in today’s health care processes. It is a common, if not the most frequent, source of health information for consumers and is an integral part of the health care decision-making process. Congruent with existing evidence on OCHI, the outcomes of using OCHI are generally positive, especially when information sources are reliable. However, negative outcomes were reported consistently in terms of tensions across this study: the literature review, consumers in stage 1, and health practitioners and librarians in stage 2.

### Online Consumer Health Information Use–Related Tensions

Elaborating on the Merriam-Webster dictionary definition of tension, the term OCHI tension refers to the feeling of uneasiness people who actively search for online health information experience with themselves, with other people, as well as vis-à-vis the health care services. Therefore, we argue that tension is a comprehensive and meaningful construct that represents a variety of negative outcomes along three dimensions. [33].

#### Level 1: Internal Tensions

These are outcomes that affect the consumer alone because of seeking and using OCHI and are associated with an emotional state. Internal outcomes uncovered in this study include increased worrying and anxiety. It has been suggested that “challenge and confusion, and dealing with the familiar and with the contradictory, are sources or triggers of emotional behavior in information situations” [33]. Moreover, with some consumers’ lack of theoretical knowledge and ability to critically evaluate the information, this will inevitably lead to misinterpretation and unnecessary fear and anxiety [34].

Consumers who have even moderate levels of health anxiety are more likely to seek higher amounts of OCHI and spend more time online for health purposes overall [35,36]. One influencing factor is the individual’s proneness to worry; one study examining the relationship between *anxiety sensitivity* and OCHI use reported that there was a relationship between exposure to OCHI and the etiology and maintenance of anxiety sensitivity [37].

#### Level 2: Interpersonal Tensions

Interpersonal tensions include any strain in the relationship between the OCHI consumer and other individuals, such as their HCP or a family member. An example of how this strain in the patient-clinician relationship occurs is when the practitioner does not acknowledge or validate the information brought in by their patient. A lack of trust developed when patients found information online that their HCP had not mentioned during the clinical encounter. This has also frequently been reported in the literature: patients who have read health information online may give less credence to their doctor’s opinion and may use the information to test their doctor’s knowledge, causing damage to the patient-clinician relationship [34,38]. On the other hand, some doctors lack the communication skills or are not up to date on all the information available and thus, report difficulties in dealing with OCHI.

#### Level 3: Service-Related Tensions

These tensions refer to any strain in the relationship between an OCHI consumer or patient and the health care system, leading to a change in the individual’s use of health care services or adherence to management plans. This is in line with results reported in other studies. In one study, over 11% of the respondents reported that finding health information online led to them refusing or discontinuing treatment recommended by a physician or dentist [39]. Other studies also reported that participants (35% and 29.9%) would use the internet as a health information source instead of getting a professional opinion [40,41]. On the other hand, it was also reported that OCHI could lead to more frequent encounters with their HCP based on the information found [41].

**Figure 2:**
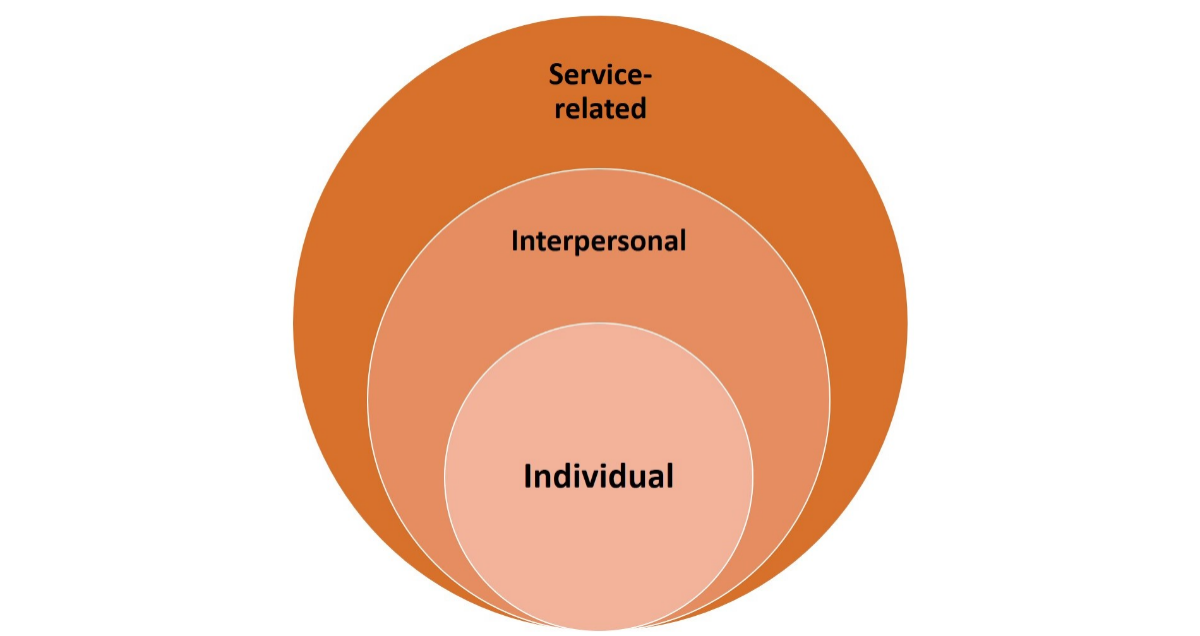
Online consumer health information (OCHI) use–based tensions.

On the basis of this construct of tension, we conceive the *OCHI use–related tensions* as presented in Figure 2. This enriches our original conceptual framework and adds to the scientific knowledge on the outcomes of OCHI use [15]. In the literature, there is an established link between health anxiety (internal tension) and the patient-clinician relationship (interpersonal tension). Health anxious people are more prone to wrong self-diagnosis and unnecessary worries, which is likely to increase the risk of misunderstanding and frustration with their doctor [35]. They may also feel that the duration of the clinical encounter was not enough to discuss all their worries and be less satisfied with the consultation [32]. Moreover, there are studies that report a link between low trust in the physician (interpersonal tension) and nonadherence or mal-adherence to a management plan (service-related tension) [42]. Our results suggest there is a relationship between the three dimensions of tension.

### Potential Preventive Strategies

**Figure 3:**
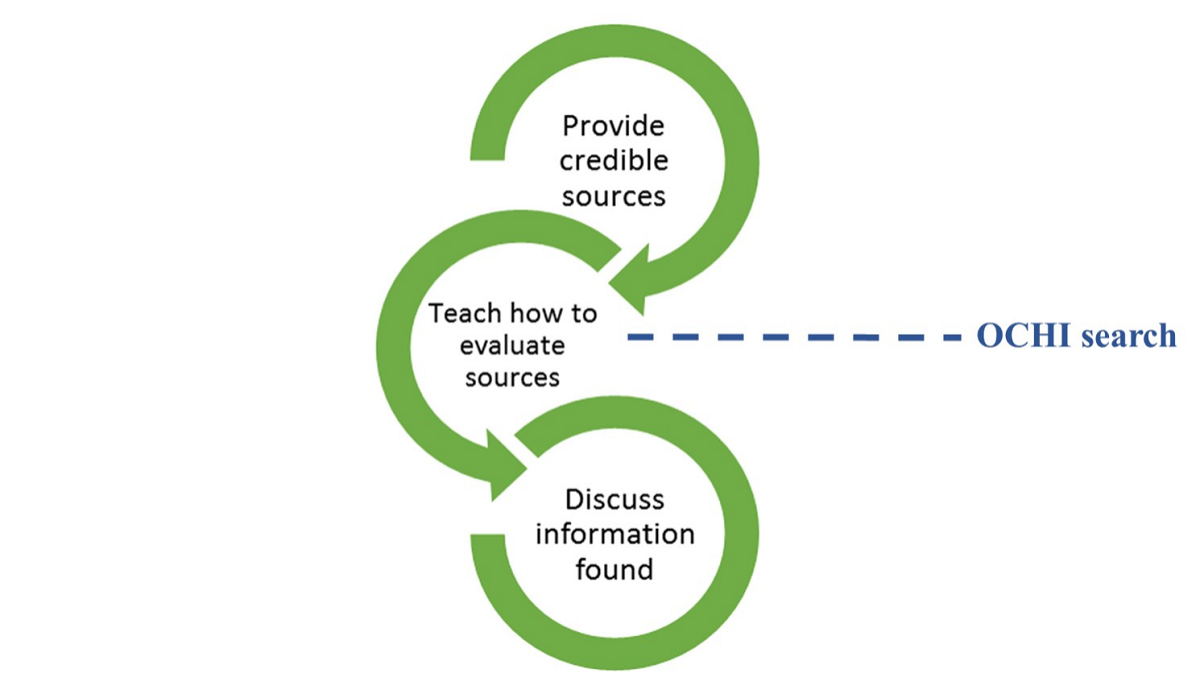
Strategies to reduce online consumer health information (OCHI) tensions.

Several strategies targeting different periods of the iterative information-seeking process have been identified: before seeking the information online, while searching for information online, and after finding the information online. They can be summarized into three main preventive strategies as shown in Figure 3. During data analysis, it became clear that the health librarian participants in the second stage provided a distinct separate viewpoint and played a very different role from health practitioners.

#### Before Online Consumer Health Information Search: Providing Reliable Sources

Health practitioners preemptively provide patients with the names of reliable, reviewed websites during the clinical encounter rather than wait for patients to navigate on their own. This is in line with other studies that recommend that HCPs, specifically doctors, guide patients to reliable sources of OCHI [34,43-45]. Inevitability, people will try to search for health information online; however, they may not be adequately equipped to deal with the vast number of OCHI resources. In one study, even physicians expressed a need for training on how to navigate OCHI resources so that they are better able to recommend websites to their patients [46].

#### During the Search: Teaching Consumers How to Evaluate Online Consumer Health Information Sources

In the literature, it has been reported that evaluation interventions led to a more critical evaluation of online information [47]. This education process, however, is time-consuming and may not be a priority during the clinical encounter. Although practitioners could provide their patients with a list of criteria for reliable websites, there are also online resources available in the form of guidelines and checklists to follow while evaluating a website. However, many consumers, especially those in a lower socioeconomic strata, may not be aware of these resources or the fact that they are not correctly evaluating resources [48]. As suggested by participants in this study, an organizational effort is needed, for example, through mass media, in school curriculums, or in public libraries.

#### After Finding Relevant Online Consumer Health Information: Discussing the Information Found

The final strategy is discussing the information found with a health professional (eg, someone in their social network or a nurse phone line). This is supported by the literature; it was reported that patients simply need to have the information they found explained, contextualized, or validated by a health practitioner [34,40]. Studies report that discussing the information they had found with their physicians had a positive effect on the patient-clinician relationship, led to more involvement in decision making, and led to reduction of worries [32].

For health practitioners, there are ongoing initiatives to add OCHI into their continuing education (eg, workshops on dealing with their informed patients). For consumers, there are initiatives to encourage them to discuss information with their providers either through the help of a health librarian who can help organize the information and questions, or applications and websites that aid in that role (eg, the webpage Discutons Sante). There are, however, limitations to discussing OCHI; time limitations during the clinical encounter, and there may be a barrier related to practitioner attitudes. There may also be a barrier in understanding the health information (because of low health literacy or low education) or a limited social network.

#### The Key Role of Librarians

Librarians are responsible for providing reliable health information and advocating the advantages of using OCHI for informed decision making. Working with consumers and health practitioners, they are well positioned to implement the preventive strategies described in this study. The integration of health librarians into the consumer’s health information–seeking process may help ensure the reliability of the OCHI they find and use, as well as the appropriateness of its level of health literacy, leading to fewer internal tensions. Librarians’ involvement may facilitate the discussion with health practitioners, leading to fewer interpersonal tensions. Finally, they can help consumers find situationally relevant OCHI, helping them to make more appropriate health care decisions and potentially leading to fewer service-related tensions.

Two barriers to the integration of health librarians into this information-seeking process should be noted: the lack of awareness of available health librarian services and the lack of access to health librarians by the public (because of their location inside hospitals). One potential solution would be to train community librarians working in public libraries on how to provide health information services and instruction, or at least train them to refer consumers to the local hospital-based health librarian.

### Strengths and Limitations

Most respondents in stage 1 were females in the age range of 18 to 24 years. Although the lack of heterogeneity of our sample may present a limitation, studies report that the majority of individuals who search for and used OCHI are young women, which is reflected in our sample [40,49]. A recent systematic review on the use of Facebook in recruiting participants for health research purposes suggests that using social media to recruit participants may have led to this young female population sample [25]. A future study could use other recruitment tools to focus on an older population and to explore differences. One strength of our study is that our participants were all key informants, purposefully sampled for their experience and knowledge on the topic, as well as their willingness to incorporate OCHI in their practice [27,50]. No new ideas emerged in the final few interviews, and there was corroboration after triangulation of results of the review and all interviews.

### Conclusions

The purpose of this investigation was to describe the negative outcomes associated with using online consumer health information, as well as to identify and reflect on any potentially preventive strategies.

This work makes two major contributions to the advancement of knowledge on OCHI. The first concerns a fine-grained identification of OCHI negative outcomes, which results from the construct OCHI use–related tension with three different and noninclusive levels of tensions (dimensions): individual, interpersonal, and service-related. This original construct enriches the original conceptual framework on outcomes of OCHI use and can serve as a foundation for future research. The second contribution, which involves clear practical implications, refers to the strategies primary care consumers, community, and health librarians and all types of primary care practitioners could adopt to prevent the risks associated with OCHI use. Exploration of these strategies and their implementation will be part of our future research.

## Appendix

Multimedia Appendix 1 Survey used as a recruitment tool.

Multimedia Appendix 2 Interview guides.

## Abbreviations

HCP: health care provider
OCHI: online consumer health information
