# Supplementary figures and images for: Reducing Negative Outcomes of Online Consumer Health Information: Qualitative Interpretive Study with Clinicians, Librarians, and Consumers
Source: J Med Internet Res. 2018 May 4;20(5):e169. doi: 10.2196/jmir.9326 (PMC5960043; doi:10.2196/jmir.9326)

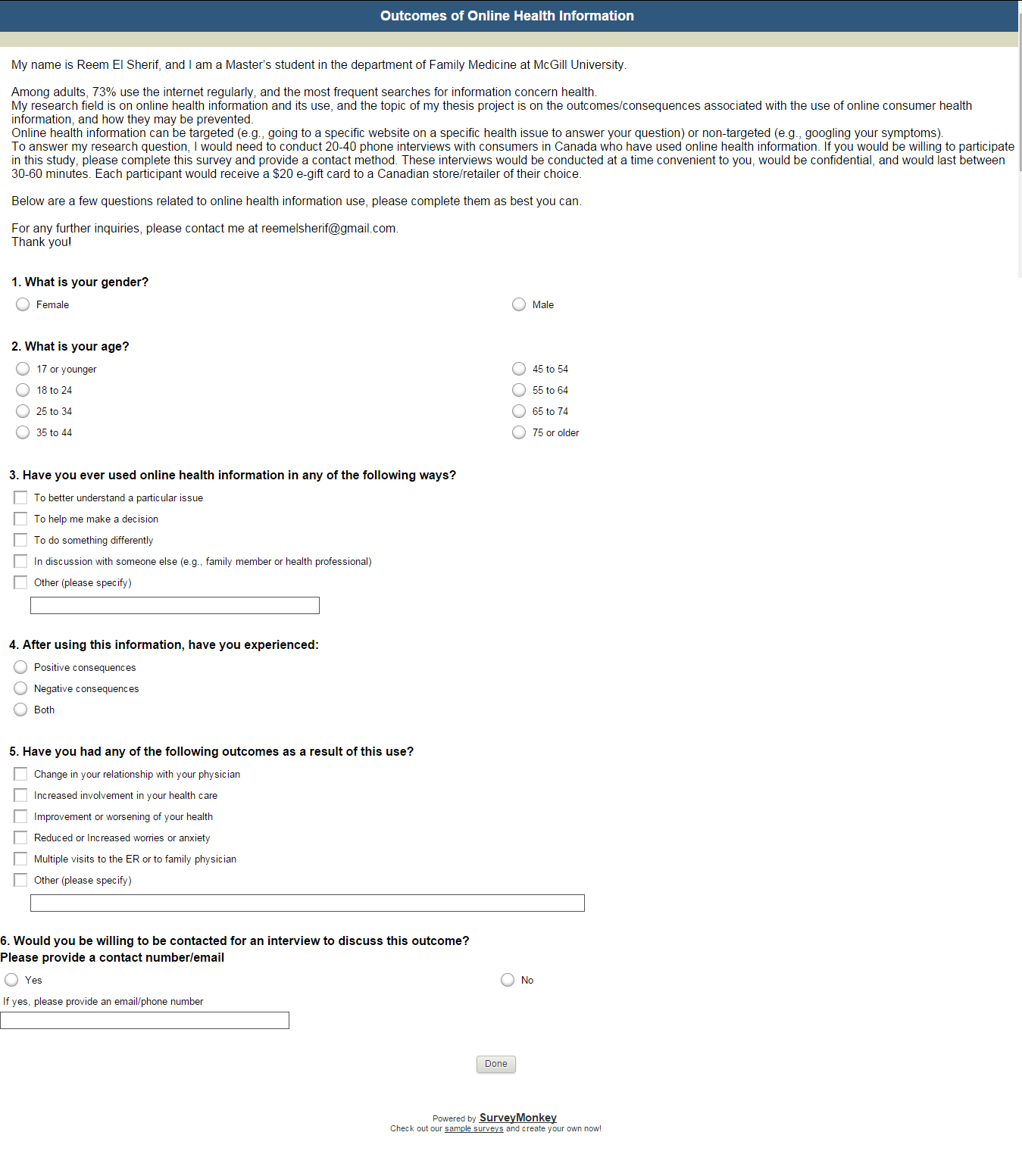

Supplement: Multimedia Appendix 1 [file jmir_v20i5e169_app1.png]
