# Supplementary material for: Reducing Negative Outcomes of Online Consumer Health Information: Qualitative Interpretive Study with Clinicians, Librarians, and Consumers
Source: J Med Internet Res. 2018 May 4;20(5):e169. doi: 10.2196/jmir.9326 (PMC5960043; doi:10.2196/jmir.9326)
Supplement: Multimedia Appendix 2 [file jmir_v20i5e169_app2.pdf]

Appendix II. Interview Guides

Interview with consumers in Stage 1

Introduction

Thank you for agreeing to being contacted for this interview. You may recall, you recently answered a few questions on the use of online health information on an online survey. You had stated that you had experienced a negative outcome of some sort after using online health information, and that experience will be the subject of this interview.

I am a Master's student in the department of Family Medicine at McGill University, and the topic of my thesis project is the negative outcomes associated with the use of online consumer health information, and how they may be prevented.

I want to state that this interview is completely confidential, and that I will be recording it to transcribe later, but all personal details will be omitted in the analysis

Before we start, do you have any questions?

Screening Question

Are you currently on any medication or being treated for any long-term disease?

Questions on information-use

1) When do you seek health information online?
2) Could you elaborate on the circumstances that lead you to look for health information online?
3) How did you look for the information?
4) (Prompt: Could you describe the process?)
5) Did you find information you were looking for?
6) If not, why do you think you did not?
7) If you did find the information, did you understand it/did it make sense to you?
8) If not, why not?
9) How did you use the information?

Questions on negative outcomes

10) After using the information, what outcomes did you experience? (What happened next?)
11) Would you describe any of these outcomes as negative?
12) In what way?
13) May you describe what happened?
14) How do you think these negative outcomes could have been avoided?

15) Are there any other details you would like to add/mention?

Thank you very much for your time.

Interview with health practitioners and librarians in Stage 2

Introduction

Thank you for agreeing to being contacted for this interview. You had stated that you had experience with consumers/patients who had experienced a negative outcome of some sort after using online health information, and that experience will be the subject of this interview.

I am a Master's student in the department of Family Medicine at McGill University, and the topic of my thesis project is the negative outcomes associated with the use of online consumer health information, and how they may be prevented.

I want to state that this interview is completely annonymous, and that I will be recording it to transcribe later, but all personal details will be omitted in the analysis.

Before we start, do you have any questions?

At an earlier stage in the project I had interviewed consumers who had experienced negative outcomes, and developed 3 vignettes from their stories. Could you please read these vignettes before we proceed?

Questions on consumer information-use and negative outcomes

1) What do you think of these stories?
2) Do you believe these are common outcomes of information use?
3) Have you had experience with these negative outcomes?
4) Could you elaborate on your experience with consumers who have used online health information?
5) Could you describe the negative outcomes associated with this use?
6) What factors do you believe contributed to the development of these negative outcomes?
7) How do you think these negative outcomes could have been avoided?
8) What factors do you believe led to these negative outcomes?
9) *If the vignette involves a practitioner/librarian* What would you have done differently?
10) *Only with physicians* How do you view patients who seek online health information?

11) Are there any other details you would like to add/mention?

Thank you very much for your time.
